# Population Mobility and Spread of HIV Across the Indo-Nepal Border

**Published:** 2007-09

**Authors:** Binod Nepal

**Affiliations:** National Centre for Social and Economic Modelling, University of Canberra, ACT 2601, Australia

**Keywords:** Acquired immunodeficiency syndrome, Disease outbreaks, HIV, Injecting drug use, Migration, Sex behaviour, India, Nepal

## Abstract

The article reviews information on the epidemiology of HIV/AIDS and behavioural networking to examine the role of population mobility in spreading HIV across the Indo-Nepal border. Documents were collected through a systematic search of electronic databases and web-based information resources, and the review focuses on studies about types of the virus, prevalence of HIV, and sexual and injecting networking. HIV-1 (subtype C) and HIV-2 were identified in Nepal. The prevalence of HIV was higher among male labour migrants and female sex workers (FSWs) who returned from India, especially from Mumbai, than in similar non-migrant groups. In the early 2000s, about 6–10% of Mumbai returnee men, compared to up to 4% of India returnee men and up to 3% of non-migrant men in the far-west Nepal, were identified with HIV. Likewise, when the prevalence of HIV among sex workers in Kathmandu was found to be 17% in 1999–2000, up to 44% of India returnee and 73% of Mumbai returnee FSWs were identified with the virus. These data are, however, based on small samples with questionable representativeness of the target populations and need to be interpreted cautiously. They also generate a biased impression that HIV was coming into Nepal from India. Recently, the possibility of a two-way flow of HIV across the Indo-Nepal border through injecting and sexual networking have been indicated by serological and behavioural data from a south-eastern cluster of Nepal and a north-eastern district of India. Although similar behavioural networks exist along other segments of the border, serological data are unavailable to assess whether and how extensively this phenomenon has caused the spread of HIV. Collaborative research and interventions covering both sides of the border are desirable to fully understand and address the prospect of HIV epidemics associated with cross-border population mixing.

## INTRODUCTION

It has long been assumed that cross-border migration of adult Nepalese, especially the migrants returning from India, could significantly contribute to the HIV/AIDS epidemic in Nepal (1-5). To address this challenge, by the mid-1990s, a few prevention programmes, including cross-border collaborative interventions, were initiated (6). However, the spread of the virus continued. In 1998, Seddon reviewed the status of, and responses to, the HIV/AIDS epidemic in Nepal and speculated that AIDS was an impending crisis in this impoverished country (3). He noted, “The rapid spread of HIV-AIDS in India is of major significance for the future development of the epidemics in its small northern neighbour, Nepal, given the constant movement of people between the two countries and the necessity for the Nepalese economy to maintain these links at all levels—national, regional, and household” (3).

Until 2002, little serological and behavioural data on migrants were published (7). A number of studies conducted in 2001 and later have been made publicly available. A synthesis of these studies will provide an opportunity to empirically assess the evolving connections between population mobility and the risk of HIV across the Indo-Nepal border.

This article reviews information on the epidemiology of HIV and behavioural networking to examine the role of population mobility in spreading HIV across the Indo-Nepal border. It draws data from studies on the molecular epidemiology of the virus, serological and behavioural surveys, and qualitative studies. The article examines the evidence on types of the virus, prevalence of HIV among major subpopulations with special focus on migrants, and cross-border sexual and injecting networking. Lack of representative and longitudinal study data is still a challenge to draw any firm conclusion on the evolution of the HIV/AIDS epidemic in Nepal and the role of mobility in this epidemic. Yet available evidence suggests that interlinked epidemics are evolving along the border areas and in some inland pockets where migration is common.

## MATERIALS AND METHODS

A systematic review was conducted in May-June 2006. Information was retrieved from documents available mainly in electronic databases and on the websites of specialized agencies. This involved four stages. First, using the boolean [(HIV or AIDS) and Nepal], 116 documents were retrieved from the Medline database (www.pubmed.gov) on 16 May 2006. Second, websites of several national and international agencies were browsed. The most important being the online collections of: UN Nepal Information Platform (http://www.un.org.np/), Family Health International (http://www.fhi.org/en/HIVAIDS/country/Nepal/nepaltools.htm), and National Centre for AIDS and STDs Control (http://www.ncasc.gov.np). These sites housed a number of reports on quantitative and qualitative studies, estimates of HIV/AIDS cases, policy analyses, and government strategies. Third, a search was carried out on the website of the International AIDS Society which indexed about 120 abstracts on HIV/AIDS in Nepal. A scrutiny of the abstracts revealed that some presentations were posted on the websites of the organizations mentioned above, and a few others were published in journals (indexed in Medline). Abstracts which were poorly written and unlinked to their source reports were excluded. Fourth, the collected documents were skim read to assess whether they contained information on HIV in conjunction with cross-border mobility or cross-border sexual and injecting networking. The documents included in the review are listed in the references. Six studies provided serological data with more than one case of HIV and by migration status of the study population. Only two studies contained information on subtypes of the virus.

## RESULTS

### Mobility across the Indo-Nepal border

Nepal shares about a 1,700-km long open border with India in the East, South and West, and about a 1,200-km long controlled border with the Tibet Autonomous Region of China in the North. The ever snow-covered Himalaya range stands between Nepal and China, and the border areas are sparsely populated. The people of land-locked Nepal depend almost entirely on India for (overland) transportation to any third country. In addition, these two countries have had very strong cultural, religious, social, and economic ties since ancient times.

The people of Nepal cross the border to India for various purposes, such as work, study, trade, pilgrimage, cultural visits including marital exchanges, and the like. The people of India also visit Nepal for business, work, pilgrimage, tourism, and so on. India has long been the major destination for Nepalese, and most foreigners in the country are Indians. According to the 2001 Census of Nepal, 762,181 persons were abroad, with 78% in India (8). The Census counted 116,571 foreign citizens residing in Nepal, of whom 88% were Indians (8). Short-term and short-distance mobility, including movement of transportation workers, are also important issues in the context of HIV epidemics, but conventional population censuses and surveys do not adequately cover these issues. Census information on immigration and emigration can only provide a minimal figure of cross-border mobility.

Results of small-scale studies conducted in specific regions, districts, villages, or cities suggest that mobility across the Indo-Nepal border is very high. In a 1997 survey conducted in five border towns of Nepal, seven of 10 adult men reported that they visited India almost every month in the past year (5). This was not surprising since the survey was conducted in the towns adjoining India, and all sorts of mobility were considered. But in inner areas also, cross-border migration—seasonal migration in particular—is common. A 1994 survey conducted in 11 districts of the far-west and mid-west regions found that 15% of adults migrated seasonally to India (1). A 1999 assessment conducted in 20% of the villages of Doti—a far-western hill district where migration was common—found that at least one member of the family from half of the households had worked abroad, nearly all (94%) in India (9). A recent estimate suggests an out-migration of up to 90% of adult men in some villages of the western and far-western hills (10). Experts suggest that a decade of violent conflict has exacerbated migration, displacement, and trafficking and intensified vulnerability to HIV/AIDS (11). In general, external migration, particularly seasonal migration, is very high in the western, mid-western, and far-western regions. Destinations and durations of stay are not uniform. Nepalese migrants visit both rural, such as Punjab, Hariyana, Himanchal Pradesh, etc., and urban destinations, such as Mumbai, Delhi, etc. Although seasonal migration—about six months on average—is common, many migrants live and work in India for several years.

Nepal is a major source country of human trafficking. Although several international and national organizations are dealing with this issue, no reliable statistics are available. It is generally quoted that each year 5,000–7,000 Nepalese women and girls are trafficked or lured into brothels in India, and 100,000–200,000 Nepalese women and girls are working in Indian brothels, large proportions of them in Mumbai (3,7). Ali notes that these statistics are recycled over and over, without cross-verifications, and hence it is virtually impossible to examine the trend (12). Some Indian and Nepalese non-governmental organizations have rescued and repatriated some victims of trafficking in the past, but no statistics on the actual volume of returnees are available.

### Types of the virus

Both HIV-1 and HIV-2 are identified in Nepal, but very little is known about the sources and subtypes of the virus in various populations and parts of the country. In 2004, Chander and Pahwa reported the presence of both HIV-1 and HIV-2 viruses in the country (13). This was the first report on the evidence of HIV-2 infections in Nepal. Three of 1,534 patients screened in a teaching hospital in Bhairahawa were HIV carriers: one patient carried HIV-1 only, and the other two had both HIV-1 and HIV-2. Earlier in 2000, Oelrichs and colleagues reported HIV-1 subtype C in 36 blood samples taken from injecting drug users (IDUs) in Kathmandu (14). They speculated that the virus, possibly of Indian origin, was introduced into this population on two different occasions.

The sparse information on molecular epidemiology suggests that the HIV/AIDS epidemic in Nepal may mirror that in India where both HIV-1 and HIV-2 are present (15). This pattern is, however, not unique to India. Therefore, without further molecular epidemiological studies in various parts and populations, no conclusion can be drawn about the sources of the viruses in Nepal, for HIV/AIDS is present in multiple subpopulations in the country.

### Multiple epidemics

**Fig. 1:**
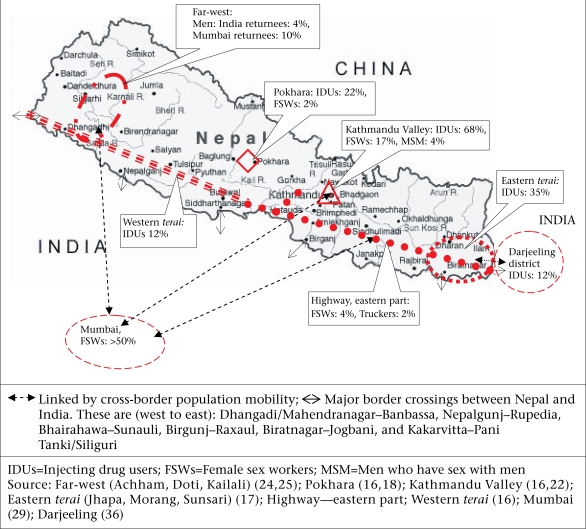
Map showing the approximate geographic locations of major HIV epidemics (according to the highest prevalence in 1999–2005) in Nepal and the major high-prevalence groups in India with which they are linked through population mobility

Despite the small geographic area and population, especially compared to India, several HIV epidemics are evolving in Nepal (Fig. 1). Available evidence suggests that IDUs and FSWs are the major behavioural groups. Men who have sex with men—with 3.9% prevalence of HIV in 2003 in Kathmandu (16)—are also emerging as a potential group at high risk, but very little is known about their distribution and behavioural networking. The prevalence of HIV among IDUs is very high in several locations. Since the mid-1990s, infections among IDUs remained consistently above 50% in Kathmandu, the capital city of Nepal. Also in Pokhara and an eastern terai cluster comprising three districts, namely Jhapa, Morang, and Sunsari, HIV is firmly established with a prevalence of 22–35% in the recent past (17,18). The epidemic is moving to the western terai regions where the prevalence of HIV among IDUs was 12% in 2005 (16). Since most IDUs are sexually active, do visit sex workers, and are quite mobile, the HIV epidemics in this subpopulation are not likely to be independent and confined to themselves. Moreover, emerging evidence on a high degree of cross-border mobility of IDUs suggest that a link might have evolved between the HIV epidemics on both sides of the border (19-21). Probably, exchange of the virus between IDUs and FSWs has occurred in Kathmandu, where the prevalence of HIV among FSWs rose to 17% by 1999 (22). This coincided with the re-emergence of HIV among IDUs. The samples of FSWs captured a few women who had returned from Mumbai brothels. It is likely that Mumbai returnee FSWs might have played a role in the reintroduction of the virus to IDU populations in the Kathmandu Valley. Outside the Valley, FSWs have low levels of HIV (Fig. 1); perhaps, the epidemics in other groups, such as IDUs, are taking time to spill over.

Although they have high levels of HIV prevalence, IDUs and sex workers together account for less than 1% of the adult population aged 15–49 years and are present mainly in cities and along highways (23). Existing in most districts, most importantly in rural areas, migrants make up the largest and the most critical population vulnerable to HIV/AIDS. About half of the estimated adult HIV cases in 2003 and 2005 were attributed to migrants (16,23).

### Migrants in focus

Results of analyses of community-based surveys, risk-group surveys, and service statistics consistently showed that men and women who had worked in Mumbai have a much higher prevalence of HIV than those who worked in other parts of India or only within Nepal. The Table shows that studies drawing samples from communities in Achham, Doti, and Kailali reported that about 6–10% of men returned from Mumbai compared to up to 4% men working in other parts of India and up to 3% of those working in Nepal were HIV-positive (24-26). This pattern of higher proportions of HIV-positive cases among Mumbai returnees than other people was reflected among men attending voluntary counselling and testing (VCT) clinics (27) and also among sex workers surveyed in Kathmandu (22,28). Of men who took services from the VCT clinics during 2001–2003, 12.5% of 32 Mumbai returnees compared to 8.5% of 106 India returnee and 2.3% of 210 internal migrants were HIV-positive (27). Although this pattern reflected the epidemic pattern seen in the far-west districts, these findings are based on clinical data and, therefore, cannot be generalized to the migrant community at large. Among sex workers surveyed in the Kathmandu Valley, HIV positivity was higher among Mumbai returnees (73%, n=12, in 2001) compared to India returnees as a whole (44%, n=9, in 1999/2000 and 42%, n=33, in 2001); and these rates were several-fold the prevalence of HIV (17% in 1999–2000 and 16% in 2000) in the overall samples of sex workers (22,28). While the total samples (300 each year) were adequate, statistics for returnee sex workers were based on very small denominators and hence prone to uncertainty. Further, it is well-known that Nepalese sex workers who are rescued, escaped, or abandoned (because of HIV-positive status) from brothels are often condemned by families and communities and hence again get involved in sex work for survival (2). However, no estimates are available about the volume of returnees and those who resume sex work. It is also less clear that where these returnee sex workers, and perhaps the migrant men, contracted the virus. Some returnees might have contracted the virus in the home country, but many of them might have returned with the virus. The returnees reflected the situation of Mumbai where HIV infections among FSWs increased sharply from 1% in 1989 to 51% by 1993 and stayed above this level thereafter (29). No information was available on differentials in the prevalence of HIV by country of origin of sex workers in Mumbai.

**Table:** Percentage of HIV-positive persons by migration status, Nepal

Population and location	Year	All		Internal migrants		Worked in India		Worked in Mumbai		Non-migrants	
		No.	%	No.	%	No.	%	No.	%	No.	%
Community											
Adult men, Doti (24∗)	2001 (Apr)	137	8.0	-		-		97	10.3	40	2.5
Adult men, Achham (25∗)	2001 (Oct-Nov)	610	2.3	100	3.0	242	3.7	90†	7.8	268	0.7
Adult men, Kailali (26∗)	2001 (Oct-Nov)	610	0.3	-		308	0.6	33	6.1	302	0.0
Clinic attendees											
Adult men, VCT attendees (27∗)	Sep 2001-Jun 2003	316	4.4	210	2.3	106	8.5	32	12.5	-	
Risk group											
Sex workers, Kathmandu (22∗)	1999–2000 (Nov-Feb)	300	17.3	-		9	44.4	-		-	
Sex workers, Kathmandu (28∗)	2001 (Mar-Aug)	300	15.7	-		33	41.7	12	72.7	-	

∗ Indicates reference no. in the References List

† Combines respondents who worked in Mumbai only (5/65=7.7%) and Mumbai and other places (2/25=7.8%). Of the 152 India returnees who did not work in Mumbai, 2 were HIV-positive (1.3%)

VCT=Voluntary and counselling testing

Besides identifying Mumbai as the high-risk destination, community-based studies provided some information on the prevalence of HIV by duration of migration and dates of return, enabling the timing of the start of HIV/AIDS epidemics among migrant labourers to be estimated. Results of studies from Achham, Doti, and Kailali imply that migrants who spent more than four or five years in India seem to have a substantially higher chance of contracting the virus than those who stayed for a shorter duration. The study in Doti reported that six (20.7%) of 29 men who lived in India for more than four years compared to three (4.5%) of 67 men who returned within four years were HIV-positive (24). Likewise, the prevalence of HIV was 12.8% (n=5) among 39 men in Achham who returned after five years compared to 2% (n=4) among 203 men who returned within five years (25). In Kailali, men who stayed in India for more than five years had a higher prevalence of HIV (1.5%, 1/66) than those who stayed five years or less (0.2%, 1/544) (26); but given a single HIV-positive case in each category, the difference needs to be interpreted cautiously. In addition, the Achham study showed that the prevalence of HIV (7.8%, 5/64) was higher among migrants who returned in the past year preceding the survey than those who returned in the past 2–5 years (3.6%, 4/111). No infection was detected among migrants (n=67) who returned five years before the survey. Based on this 2001 Achham study, it can be speculated that a notable scale of HIV epidemics in the Nepalese migrant communities might have began just after the mid-1990s.

Another important issue would be to identify the behavioural and demographic risk factors for infections due to HIV/AIDS among migrants. This is a much more difficult task because these studies had either small sample sizes or few HIV/AIDS cases identified. Considering the total samples, the Achham, Doti, and Kailali studies generally pointed out that age above 25 years, sex with sex workers, and inconsistent condom-use were distinct risk factors for sexually transmitted infections, including HIV. Only the Achham study provided differences in the prevalence of HIV by demographic characteristics of migrant returnees. The study found that HIV was higher among ever-married (4.1%, 9/218) than unmarried (0%, 0/24) people, those residing in inner rural areas (5.5%, 6/109) than in rural market areas (2.3%, 3/133), those with primary or less education (5.5%, 6/110) than secondary or higher education (2.3%, 3/132), and those who belonged to occupational castes (4.1%, 3/74) than the so-called upper castes, such as Brahmin and Chettri (3.6%, 6/168). Since no confidence intervals or statistical tests were provided, these differences need to be interpreted cautiously.

The spread of HIV from male migrants to their wives, sex partners, and women will be an immediate challenge in the areas where migration is high. Most studies have so far focused on male migrants, and little is known about HIV infections among women in migration-common areas. A clinical study conducted in 2001 among women of migrant communities of Kailali found only one HIV case (0.3%) among 900 women who were tested (30). Although the prevalence of HIV was very low in this sample compared to that of migrant men in Doti and Achham, as many as 11% of these women were diagnosed with one or more untreated sexually transmitted infection(s) (3). Since migrant returnees tend to have unsafe sex with their wives and other sex partners (31), infections among women are likely to rise. More migrants than non-migrants reported the involvement in risky sexual behaviours when away from home. In a 1994 survey in 11 mid- and far-western districts, 49% of male and 40% of female seasonal labour migrants reported premarital or extramarital sex when they were away (1). Recently, male migrant returnees of far-western Nepal mentioned that they learned the habit of gathering for drinking and then visiting brothels in Mumbai; back home also, they looked for extramarital affairs with local girls (31).

### Cross-border networking

In addition to the sexual networking associated with the seasonal migration of Nepalese males, cross-border sexual and injecting networking evolving along border areas merits attention. Two studies conducted in the 1990s in border towns on the Nepal side revealed the potential for substantial cross-border sexual networking (4,5). Results of these studies suggest that Nepalese and Indian men obtain sexual services from sex workers operating informally in the Nepalese towns and formally in the red light areas of India. Although no red light areas exist in Nepal, cabin restaurants and hotels supply drink and sex for customers from Nepal and India. Any adult male visiting the border towns can find several pimps approaching him to make a trip to a brothel across the border or in a lodge in a Nepalese town. Transportation workers, especially truck drivers and their assistants, are the most visible clients of sex markets operating formally and informally on both sides of the border. Every day, thousands of trucks cross the border, and truckers halt at the border-crossing for a day or more. It is not uncommon for them to solicit casual sex during this period. They are, however, not the only clients, and sexual activities are not the only risk behaviours. Businessmen, students, labourers, and others are also on the scene, and injecting drug-use is also emerging.

Studies conducted in the early 2000s near the Indo-Nepal border areas provided empirical evidence on cross-border movement of people for drink, drugs, and sex. Many sex workers operating in Nepal, especially in the areas bordering India, have a history of sex work in Indian sex markets. In 2003, more than 62% of 400 FSWs interviewed in Kathmandu were selling sex in this city for less than a year; about 8% reported their involvement in sex work outside the Kathmandu Valley, and about 2% had sold sex in India (32). In a 2001 survey of 400 FSWs operating along the eastern segment of the East-West highway (which runs through cities and market paces near the Indo-Nepal border) covering 16 *terai* districts, 61.5% reported that they went to other locations to sell sex in the past two years preceding the interview; about 13% of the respondents had sold sex in India (33).

Important additional information was obtained on the cross-border injecting networking from studies conducted on the use of injecting drugs on the Nepal side of the border in the early 2000s. Many Nepalese IDUs crossed the border to India to purchase drugs or to receive injections from drug dealers or pharmacists (19,34,35). This practice was very common among drug users of the *terai* towns which are close to Indian border towns (in the eastern *terai*, for example, Biratnagar is about 3 km and Dharan is about 50 km from Jogbani). In a 2003 survey of 345 IDUs in the eastern *terai* cluster, a large majority of respondents mentioned a history of having injections not only in other locations of Nepal but also in India (17). In this study, 85% of respondents stated that they had injected drugs in places away from their usual place of residence in the past year. Most of them had taken drugs by injection in various parts of India. The list of Indian towns where these IDUs had been in the past year included places as close as Jogbani, Pani Tanki, and Siliguri to as far as Mumbai, Delhi, and Nagaland. Most frequently cited were the adjacent towns, such as Jogbani (n=212) and Pani Tanki (n=23) (Annexure 13 (17)). Mobile IDUs were more likely to be HIV-positive than non-mobile IDUs (odds ratio=3.5; 95% confidence interval [CI] 1.5–8.5) (Table 7.4 (17)). Of 292 IDUs who went to other parts of Nepal or to India, a large minority exchanged contaminated injecting equipment with injectors at their destinations—21% borrowed from and 28% lent to local injectors (17). Ethnographic studies also showed that drug users from Kakarvitta and Birtamod (on the Nepal side) used to go to Pani Tanki to obtain drugs and injections, and they also shared injections with Indian IDUs met in those towns (35). Drug injectors of Biratnagar commuted to Jogbani (adjoining Indian town) where drugs or injections were readily available from drug dealers or pharmacists (19). At the places of drug dealers and other sites across the border, IDUs shared drugs and injecting equipment with other users from Indian and Nepalese towns, thereby enhancing the risk of exchanging the virus (19,35). Drug injectors of Kathmandu and Pokhara are even prepared to make a day-long bus trip to Indian border towns, such as Sunauli (across Bhairahawa) and Raxaul (across Birguj), to obtain drugs on some occasions, especially when supply of drugs is tight owing to the occasional police crackdown in Nepal.

**Fig. 2:**
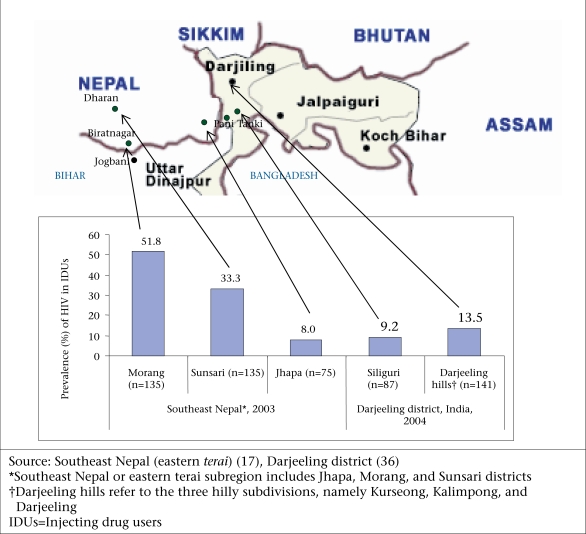
Approximate geographic locations of injecting drug-use-related HIV epidemics identified in eastern terai, southeast Nepal in 2003 and Darjeeling district, India, in 2004

Only one study on injecting drug-use and HIV epidemic was conducted in 2004 in Darjeeling district, a north-eastern district of India, sharing borders with eastern Nepal, Bangladesh, and Bhutan (Fig. 2) (36). In a sample of 228 IDUs, 11.8% (95% CI 7.9–16.7) were HIV-positive: 13.5% (95% CI 8.3–20.2) in hill areas and 9.2% (95% CI 4.0–17.3) in Siliguri, the plain area of the district. Siliguri is the most important hub city for travel and trade among Nepal, Bhutan, and all the north-eastern Indian states, including Manipur and Nagaland (injecting drug-use and HIV epidemics were widespread by the late 1980s in these two states). The authors of the Darjeeling study speculated that Indo-Nepal cross-border population mixing for drug injections and commercial sex could have introduced HIV among IDUs of this district. This speculation seems plausible because an HIV epidemic started earlier and stayed higher among IDUs on the Nepal side of the border, 35% in 2003 and 31.6% in 2005 (16,17), than on the Indian side, 11.8% in 2004 (36). The cross-border injecting networking documented by the studies on the Nepal side (17,35) (preceding paragraph) also supports this possibility. But spatial differentials showed that the prevalence of HIV was almost equal among IDUs in Jhapa (8%) and Siliguri (9.2%)—the two nearest cross-border locations (Fig. 2). No study simultaneously examined both sides of the border to determine the extent to which these two adjoining epidemics have been fuelling each other.

## DISCUSSION

The review provided an opportunity to examine and update the HIV epidemics evolving across the Indo-Nepal border. The studies conducted in the early 2000s were particularly important to draw insights on both serological and behavioural issues. There are still some areas of improvement for a better understanding of the changing situation.

First, serological and behavioural data on migrants are available from limited areas. For example, while the available studies identified Mumbai as the highest-risk destination for male migrants and female sex workers, the corresponding prevalence data were mostly based on very small denominators and samples from a few geographic areas. Also, confidence intervals around those point estimates were rarely provided. Migration is common in many other areas of Nepal. Both serological and behavioural data from other high-migration regions are needed to understand the country-wide situation of this increasingly vulnerable population. It is understandable that serological studies are difficult to conduct for various technical, ethnical and resource constraints. Nevertheless, to arrive at a definitive conclusion, future studies are expected to draw adequate and more representative samples and provide basic statistics than most past studies did. That would also enable the categories of migrants likely to be more susceptible to HIV to be assessed.

Second, the studies conducted on either side of the border were prone to biases in drawing conclusion on the roles of cross-border population mixing in the spread of HIV. The studies collecting data from only one side of the border lacked comparative perspectives of the other side. These limited cross-sectional studies are also inadequate to confirm how much infection is associated with population mobility and in which direction the epidemic is spreading. Collaborative studies (or a surveillance mechanism) that cover both sides of the border simultaneously are needed to determine the extent of cross-border exchange of HIV.

Third, since previous studies focused on male labourers and female sex workers, future research should include other significant groups, such as female labourers and Nepalese men (or Gorkhas) serving in the Indian army. Also, this article only examined HIV epidemics with reference to Indo-Nepal cross-border mobility. Rapidly-growing populations of migrants to the Middle East and Southeast Asia, for which virtually no such information is available, merit an inclusion in future studies.

Fourth, although mobility is playing and continues to play significant roles in fresh introductions of HIV in various locations, large-scale spread of the virus at local levels will ultimately depend on the risk-networks within the community. Therefore, the need for a study of sexual behaviour in the male and female population in general, as was pointed out by Furber and colleagues (7) in 2002, persists to be an unmet research need.

Finally, as pointed out by a reviewer of this article, the need for sociological and health-promotion studies have increased with the growth of the epidemic. Seddon highlighted potential roles of several issues, such as poverty, gender inequality, weak healthcare infrastructure, and human rights in his 1998 paper which depicted the HIV/AIDS epidemic in Nepal as a ‘coming crisis’ (3). The roles of such contextual factors have to be revisited in light of the sociopolitical transition taking place in the country. A detailed discussion on these issues was beyond the scope of this article, but acknowledging their importance, three issues of special significance are pointed out here. First, the nature and scale of population mobility might have changed with the civil conflict which began in 1996 and covered most parts of the country by the late 1990s. In consequence, displacement of population rose, healthcare systems crumbled, and vulnerability to HIV/AIDS increased (11).

With the signing of the peace accord between the Government and the rebel forces in November 2006, migrants and displaced persons can be expected to return home. Will this pleasant development contribute to increased sexual mixing and perhaps the spread of HIV? Are there healthcare systems or prevention programmes ready to provide basic awareness and preventive methods? Second, in the 1980s and 1990s, stigma and indifference to people with HIV/AIDS were based partly on the misperception that AIDS was a foreign or Western disease. Although this notion has been vanishing, another misunderstanding that AIDS is a Mumbai disease is taking hold (24). How does this new misperception affect prevention and care practices? Third, the view that HIV is flowing from India to Nepal cannot be sustained in light of emerging evidence which indicates a possibility of a two-way flow along the border areas. Unlike most other international borders, the Indo-Nepal border allows unrestricted movement of their citizens. Yet surveillance and interventions, including awareness programmes, depend on polices and resources of the respective countries. In such a situation, how can cross-border collaboration between governments and among non-government agencies be effectively instituted to reach the mobile populations with surveillance, educational messages, and prevention and care programmes?

## CONCLUSION

Contrary to past assumptions in Nepal that trafficked women and migrant men bring HIV from Indian cities, the spread of HIV across the Indo-Nepal border is perhaps evolving as a two-way process. Serological evidence indicates that Nepalese men and women were bringing HIV to Nepal from India. This is partly due to the study designs that only involved non-migrants and migrant returnees in Nepal. Little information is available to show that Indians who returned from Nepal and HIV-positive Nepalese nationals transmitted the virus to Indians. Information on mobility and behavioural networking of sex workers and IDUs suggest that the transmission of HIV is likely to occur as a two-way process. This might contribute to consolidating an epidemic zone along the border, involving both injecting and sexual routes of HIV transmission. This potential epidemic zone is perhaps already linked to the large Asian belt of drug-use and HIV epidemics originating from the Golden Triangle and connecting Viet Nam in the east to Manipur, India, in the west (37). It will be too costly to ignore this evolving epidemic just as a cross-border transmission of limited scope.

Published information is still inadequate to draw any firm conclusion on the extent, scope, and potential of HIV epidemics linked to population mixing along the Indo-Nepal border. Further collaborative and comparative studies are needed to examine injecting and sexual behaviours on both sides of the border. The focus of such studies should be not only seasonal migrants of far-western Nepal and trafficked women, but also short-term and short-distance mobility of residents and visitors along the border areas and semi-permanent groups, such as Gorkhas serving in the Indian army. Programmatic interventions should be collaborative and should cover short-term and long-term, and short-distance and long-distance mobile populations on both sides of the border areas.
